# Reduced expression of Axin correlates with tumour progression of oesophageal squamous cell carcinoma

**DOI:** 10.1038/sj.bjc.6600941

**Published:** 2003-05-27

**Authors:** M Nakajima, M Fukuchi, T Miyazaki, N Masuda, H Kato, H Kuwano

**Affiliations:** 1 Department of Surgery I, Gunma University Faculty of Medicine, 3-39-22 Showa-machi, Maebashi, Gunma 371-8511, Japan

**Keywords:** Axin, oesophageal squamous cell carcinoma, carcinogenesis, immunohistochemistry, single nucleotide polymorphism

## Abstract

Axin is a negative regulator of the Wnt signalling pathway, and genetic alterations of *AXIN1* have been suggested to be an important factor of carcinogenesis in some tumours. The objective of this study was to clarify the clinicopathologic and prognostic significance of Axin in oesophageal squamous cell carcinoma (SCC). Immunohistochemical staining for Axin was performed on surgical specimens obtained from 81 patients with oesophageal SCC. Western and Northern blottings were performed on proteins and RNA from oesophageal SCC cell lines. Then polymerase chain reaction–single-strand conformational analysis (PCR–SSCP) was performed on DNA from oesophageal SCC patients and cell lines. Axin expression was found to be correlated inversely with depth of invasion, lymph node metastasis, and lymphatic invasion. Although univariate analysis showed Axin to be a negative predictor, multivariate analysis showed that it was not an independent prognostic marker. In all but one of the seven cell lines examined, the levels of protein expression were equivalent to RNA expression. PCR–SSCP showed that five patients and three cell lines had polymorphisms in exon 4 or 5 of the *AXIN1* gene, but none of the 81 patients with oesophageal SCC had mutations. Our findings suggest that reduced expression of Axin is correlated with tumour progression of oesophageal SCC. However, additional studies will be necessary to elucidate the mechanism responsible for loss of Axin expression in tumour cells.

The Wnt signalling pathway regulates cellular proliferation, differentiation, morphology, and motility in vertebrates and invertebrates (Zeng, 1997; Akiyama, 2000; Bienz and Clevers, 2000). Axin, a negative regulator of this pathway, promotes phosphorylation of serine/threonine in exon 3 of *β*-catenin by forming a complex with adenomatous polyposis coli (APC) and glycogen synthase kinase-3*β* (GSK-3*β*) (Ikeda *et al*, 1998, 2000; Kishida *et al*, 1998). Phosphorylated *β*-catenin is quickly degraded via a ubiquitin–proteasome pathway in the cytoplasm (Nakamura, 1997). Upon Wnt signalling, because the activity of Axin complex is blocked through Dishevelled, phosphorylation of *β*-catenin is suppressed and *β*-catenin accumulates in the cytoplasm. Accumulated *β*-catenin protein is translocated to the nucleus as a coactivator for the T-cell factor (TCF)/lymphocyte enhancer-binding factor (LEF) family (Morin *et al*, 1997; He *et al*, 1998) and activates the transcription of Wnt target genes such as c-myc (He *et al*, 1998) or cyclin D1 (Tetsu and McCormick, 1999).

The wild-type Axin gene (*AXIN1*) is regarded as a tumour suppressor in some kinds of tumours. *AXIN1* mutations have been reported in a colon carcinoma cell line (Webster *et al*, 2000), hepatocellular carcinoma (HCC) (Satoh *et al*, 2000; Laurent-Puig *et al*, 2001), ovarian endometrioid adenocarcinoma (Wu *et al*, 2001), and sporadic medulloblastoma (Dahmen *et al*, 2001). In HCC cell lines with *AXIN1* mutations, accumulation of *β*-catenin in the cytoplasm or nucleus has been observed, and the transcription activity of TCF4 is regulated positively (Satoh *et al*, 2000).

Oesophageal carcinoma is one of the most lethal gastrointestinal malignancies. Despite recent advances in therapy and management, the overall 5-year survival rate remains at less than 50% (Ando *et al*, 1997; Collard *et al*, 2001). In future, it will be possible to identify prognostic markers and thus select the most suitable therapy for each tumour.

Although several studies have been performed to elucidate the relation between Axin expression and tumours in several organs, to our knowledge, there have been no reports related to immunohistochemical expression of Axin in oesophageal carcinoma, or the association between Axin expression and prognosis.

To clarify whether Axin expression is a significant prognostic factor, we examined immunohistochemically the relation between Axin expression, pathologic tumour variables, and prognosis in patients with oesophageal squamous cell carcinoma (SCC). Next, to clarify the mechanism of regulation of Axin expression, we performed Western and Northern blot analyses of oesophageal SCC cell lines. We also searched for mutations of *AXIN1* that were considered to activate the Wnt signalling pathway.

## MATERIALS AND METHODS

### Patients

Surgical specimens were obtained from 81 patients (70 males and 11 females) with oesophageal SCC, who underwent potentially curative surgery at the Department of Surgery I, Gunma University Faculty of Medicine, between 1983 and 2000. The age range of the patients was 40–78 years, and the mean age 61.3 years. Tumour stage and disease grade were classified according to the fifth edition of the TNM Classification of the International Union Against Cancer (UICC). None of the patients had received irradiation or chemotherapy before surgery, nor did any of them have haematogenic metastases at the time of surgery. Patients who underwent noncurative surgery and/or had inadequate follow-up were not included in the study. Postoperative chemotherapy and/or radiation therapy were not performed until recurrence of the tumour was confirmed by radiologic or endoscopic examination. All patients signed informed consent forms according to our institutional guidelines.

### Cell culture

Seven human oesophageal SCC cell lines were grown on plastic tissue culture dishes: TE-series 1, 2, 8, 13, and 15 (gift from Dr T Nishihira, Tohoku University, Sendai, Japan) (Nishihira *et al*, 1993), and TT and TTn (JCRB0262 and 0261, gift from Dr K Takahashi, Tohoku University, Miyagi, Japan). The TE-series were cultured in RPMI 1640 medium (Sigma, St Louis, MO, USA) containing 10% foetal bovine serum and antibiotics (100 U ml^−1^ penicillin and 100 *μ*g ml^−1^ streptomycin); TT and TTn were cultured in a 1 : 1 mixture of Dulbecco's modified Eagle medium and Ham's F-12 medium (Sigma) containing 10% foetal bovine serum and antibiotics, as described above.

### Immunohistochemistry for Axin

Resected specimens were fixed with 10% neutral-buffered formalin and embedded in paraffin blocks. Sections, 4 *μ*m thick, were deparaffinised with xylene, rehydrated, and incubated with fresh 0.3% H_2_O_2_ in methanol for 30 min at room temperature. After rehydration through a graded ethanol series, tissue sections for the Axin study were autoclaved in 20 mM citric acid buffer at 120°C for 2 min and then cooled to 30°C. After incubation with normal goat serum (Histofine SAB-PO (R) kit; Nichirei, Tokyo, Japan), the tissue sections were applied for 30 min and removed by blotting. The sections were then incubated overnight with primary rabbit anti-Axin polyclonal antibody (Zymed Laboratories Inc., San Francisco, USA) at a dilution of 1 : 100 in PBS containing 1% bovine serum albumin at 4°C, washed in PBS, and incubated with secondary antibody for 30 min at room temperature. Immunohistochemistry was performed with the SAB-PO (R) kit. The chromogen was 3,3′-diaminobenzidine tetrahydrochloride, applied as a 0.02% solution containing 0.0055% H_2_O_2_ in 50 mM Tris-HCl buffer, pH 6.0. The sections were lightly counterstained with haematoxylin. Negative controls were prepared by substituting normal rabbit serum for each primary antibody, and no detectable staining was evident.

### Evaluation of Axin expression

The mean Axin expression rate in the 81 primary tumours was almost 50%. Therefore, when 50% or more of the tumour cells in a given specimen were positively stained to the same degree as normal epithelium, the sample was graded as Axin preserved. When fewer than 50% of the tumour cells were stained to the same degree as normal epithelium, the sample was graded as having reduced expression.

### Western blot analysis

Protein extraction and immunoblotting were performed as described previously (Kain *et al*, 1994). Lysates from exponentially growing cell lines were prepared in a buffer containing 20 mM Tris-HCl, pH 7.5, 150 mM NaCl, 1% Nonidet P-40, 1% aprotinin and 1 mM phenylmethylsulphonyl fluoride. The protein concentration was determined with a BCA Protein Assay Kit (Pierce, Rockford, IL, USA). In all, 30 *μ*g of protein from each cell line was resuspended in sodium dodecyl sulphate (SDS) sample buffer (100 mM Tris-HCl, pH 8.8; 0.01% bromophenol blue; 36% glycerol; 4% SDS) containing 1 mM dithiothreitol, boiled for 5 min, and subjected to 5–20% Ready Gels J (Bio-Rad, Tokyo, Japan). Proteins were electrotransferred to a Hybond enhanced chemiluminescence nitrocellulose membrane (Amersham Pharmacia Biotech, Buckinghamshire, UK). Proteins were immunoblotted with anti-rabbit Axin antibody (Zymed Laboratories Inc.), and bands were detected using an enhanced chemiluminescence detection system (Amersham Pharmacia Biotech). For reblotting, membranes were stripped according to the manufacturer's protocol. Anti-*β*-actin (Sigma) antibody served as the control.

### Northern blot analysis

Total RNA was extracted from the cells with Trizol Reagent (Gibco BRL, Rockville, MD, USA). In all, 20 *μ*g of RNA per lane was electrophoresed in 1.2% agarose gels containing 2.2 mol l^−1^ formaldehyde, and blotted onto a Biodyne B membrane (Pall, Tokyo, Japan). The cDNA probe was labelled using a Random Primer DNA Labelling Kit (Roche Molecular Biochemicals, Mannheim, Germany) and [*α*-^32^P]dCTP (Amersham Pharmacia Biotech). The rabbit Axin probe was digested from pcDNA3-FLAG/rAxin (full-length) (gift from Dr A Kikuchi, Hiroshima University, Hiroshima, Japan). Membranes were prehybridised at 42°C for more than 2 h and hybridised overnight at 42°C after staining with methylene blue to verify the quality and quantity of the RNA. The membranes were washed in 2 × SSC, 0.1% SDS for 15 min and 0.2 × SSC, 0.1% SDS for 15 min at 42°C. The washed membrane was exposed to X-ray film under an intensifying screen. A human 18S probe served as the control.

### DNA extraction and polymerase chain reaction–single-strand conformational polymorphism (PCR–SSCP) analysis

Small pieces of normal tissue and tumour tissue were frozen in liquid nitrogen and stored at −80°C until DNA extraction. High-molecular-weight DNA samples from seven oesophageal SCC cell lines, as well as fresh-frozen tumour and normal tissues from the 81 patients, were prepared by the phenol–chloroform method after treatment with SDS and proteinase K.

All samples were examined by PCR–SSCP analysis for mutations in exons 2–5 of AXIN1, which correspond to the binding sites of *β*-catenin and GSK-3*β*. Each exon was amplified using the nine sets of PCR primers published previously (Satoh *et al*, 2000).

Each target sequence was amplified in a 20-*μ*l reaction volume containing 10–20 ng of genomic DNA, 2 *μ*M dNTPs, 10 mM Tris-HCl, pH 8.3, 50 mM KCl, 2 mM MgCl_2_, 0.2 *μ*M each primer, 1.5 *μ*Ci of [*α*-32P]dCTP (Amersham Japan, Tokyo, Japan), and 1 U of *Taq* DNA polymerase (Applied Biosystems, Foster City, CA, USA). These samples were amplified for 35 cycles of denaturation at 95°C for 30 s, annealing at 60 or 61°C for 30 s, and extension at 72°C for 1 min. The PCR products were electrophoresed in 5% polyacrylamide with 5% glycerol gels and autoradiographed for 24 h on Kodak XAR film (Eastman Kodak, Rochester, NY, USA).

### DNA sequencing

DNA fragments were cut out of the dried gels and reamplified by PCR with the corresponding sets of primers for 40 cycles. Amplified DNA fragments were purified with a QIA quick PCR Purification Kit (QIAGEN, Hilden, Germany) and sequenced with an ABI PRISM 3100 (Applied Biosystems, Foster City, CA, USA).

### Statistical analysis

Statistical analysis was performed by the *χ*^2^ test, the Fisher exact test, and the Mann–Whitney *U*-test to assess the correlation between Axin immunohistochemical positivity and parameters. A Cox proportional hazards model for risk ratio was used to assess the simultaneous contribution of Axin expression to survival.

## RESULTS

### Immunohistochemistry of Axin

**Figure 1:**
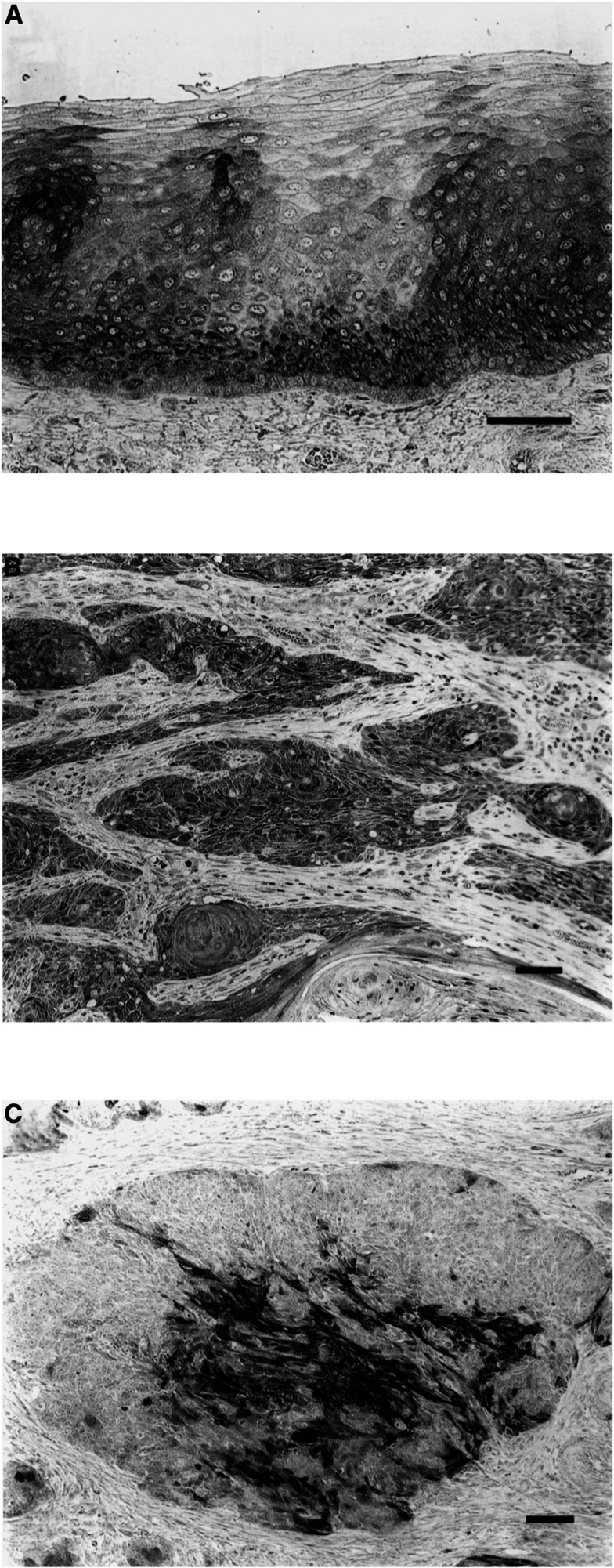
Photographs of tissue sections immunostained for Axin. (**A**) Axin was detected in the cytoplasm in normal oesophageal squamous epithelia. (**B**) Axin expression was highly preserved (Axin-preserved type). (**C**) Axin was partly detected in the cytoplasm in tumour cells, but expression partly disappeared in the peripheral cells of tumour nests (Axin-reduced type). Black bar=100 *μ*m.

Immunoreactivity for Axin was strongly positive in normal stratified squamous epithelium of the oesophagus, and was localised in the cytoplasm (Figure 1A

). Several staining patterns were observed for the expression of Axin in tumour tissues. Some tumours showed a diffuse decrease in Axin expression, others had both preserved and reduced expression in cell colonies, and others showed highly preserved expression (Figure 1B, C).

### Relation between Axin expression and clinicopathologic features

The correlation between the clinicopathologic characteristics of patients with oesophageal SCC and the expression of Axin in their tumours is summarised in Table 1

**Table 1:** Correlation between clinicopathologic characteristics and Axin expression

		**Axin expression**		
**Parameters**	***n***	**Reduced**	**Preserved**	***P*-value**
Age(mean±s.d., years)		61.3±8.5	61.9±8.6	0.7676
*Gender*				
Male	70	32	38	
Female	11	6	5	0.5853
*Location*				
Cervical	1	1	0	
Upper thoracic	10	6	4	
Mid-thoracic	50	23	27	
Lower thoracic	20	8	12	0.3218
*Grade*				
Well	21	9	12	
Moderate	39	15	24	
Poor	21	14	7	0.1497
*TNM classification*				
T				
T1	31	8	23	
T2	12	8	4	
T3	32	18	14	
T4	6	4	2	0.0235^a^
N				
N0	34	11	23	
N1	47	27	20	0.0255^a^
M				
M0	66	29	37	
M1	15	9	6	0.5136
*Stage*				
I	21	4	17	
IIA	12	6	6	
IIB	14	6	8	
III	19	13	6	
IVA	3	2	1	
IVB	12	7	5	0.0750
*Infiltrative growth pattern*				
*α*	20	6	14	
*β*	56	29	27	
*γ*	5	3	2	0.2044
*Intraepitherial spread*				
(−)	41	18	23	
(+)	40	20	20	0.5825
*Lymphatic invasion*				
(−)	25	6	19	
(+)	56	32	24	0.0058^a^
*Blood vessel invasion*				
(−)	46	20	26	
(+)	35	18	17	0.4775

s.d.=standard deviation;

a Significant.

. There were significant inverse correlations between Axin expression and depth of invasion (*P*=0.0235), lymph node metastasis (*P*=0.0255), and lymphatic invasion (*P*=0.0058). However, there was no significant association with patient age, gender, tumour location, grade, pathologic stage, intraepithelial spread, or blood vessel invasion.

As a strong inverse correlation between Axin expression and lymphatic invasion was recognised, we examined the Axin status of tumour cells that had infiltrated lymph vessels. The result revealed that most of the cases positive for lymphatic invasion had reduced or no Axin expression (50 out of 56 cases).

### Prognostic significance of Axin expression

To clarify whether Axin expression is a significant prognostic marker of patients with oesophageal SCC, univariate and multivariate survival analyses were performed. In univariate analyses by the Cox model, Axin negativity, pT classification, pN classification, pM classification, pStage, and lymphatic invasion were identified as negative predictors. In multivariate analyses, pT classification, but not Axin, was recognised as an independent prognostic factor (Table 2

**Table 2:** Univariate and multivariate analysis of Axin expression and pathologic factors

**Factors**	**Hazard ratio (95% CI^a^)**	***P*-value**
*Univariate*		
Axin	2.018(1.031–3.950)	0.0405^b^
Gender	0.483(0.147–1.581)	0.2288
Age	0.996(0.958–1.035)	0.8206
Differentiation	1.265(0.571–2.799)	0.5624
pT classification	8.245(2.889–23.527)	<0.0001^b^
pN classification	3.204(1.447–7.094)	0.0041^b^
pM classification	3.336(1.510–7.371)	0.0029^b^
pStage classification	8.338(1.996–34.842)	0.0036^b^
Lymphatic invasion	2.591(1.127–5.956)	0.0250^b^
Blood vessel invasion	1.579(0.807–3.093)	0.1824
		
*Multivariate*		
Axin	1.352(0.656–2.784)	0.4137
pT classification	5.930(1.361–25.848)	0.0178^b^
pN classification	1.503(0.488–4.628)	0.4778
pM classification	1.510(0.621–3.671)	0.3636
pStage classification	1.031(0.117–9.062)	0.9783
Lymphatic invasion	1.374(0.479–3.940)	0.5549

a CI=confidence interval.

b Significant.

).

### Expression of Axin at the protein level in cultured cells

**Figure 2:**
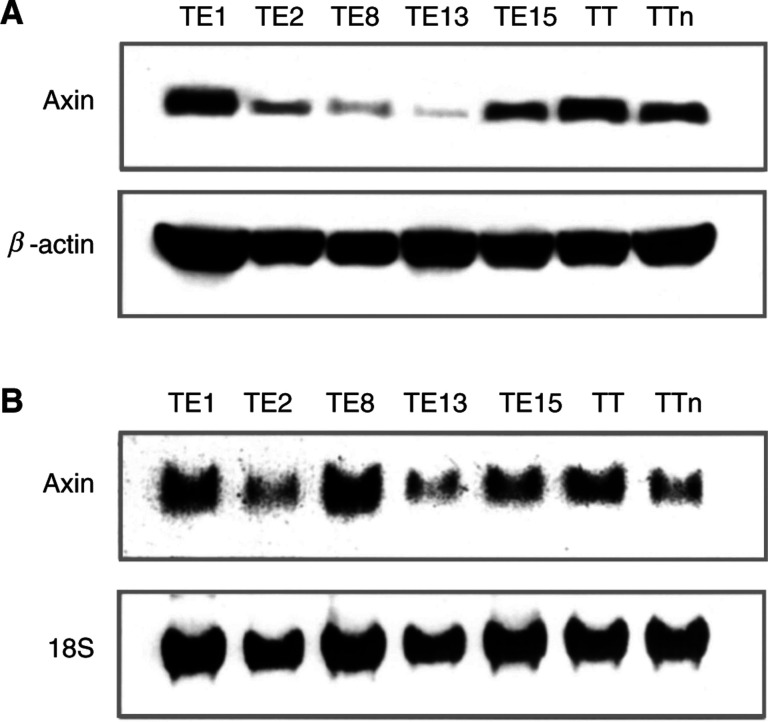
Western and Northern blotting of Axin in human oesophageal SCC cell lines. (**A**) Expression of Axin protein (top) and *β*-actin (bottom), as determined by Western blotting in various carcinoma cell lines. *β*-actin protein levels were used as controls for sample loading. (**B**) Expression of Axin mRNA as determined by Northern blotting. Top is Axin and bottom is 18S. mRNA expression is equivalent to protein expression except in the TE8 line. 18S was used as a control.

Expression of Axin was characterised at the protein level in seven oesophageal SCC cell lines. Although all of these seven cell lines were originally derived from oesophageal SCC, Western blotting revealed different levels of Axin expression (Figure 2A

). Axin was expressed at high levels in TE1, TE15, TT, and TTn, and there was very weak expression in TE2, TE8, and TE13.

### Expression of Axin at the mRNA level in cultured cells

As there were marked variations in the level of expression of Axin protein in the cultured cell lines, Northern blotting was performed to examine the underlying mechanisms of the effects of Axin on tumour cell regulation. This analysis indicated that levels of mRNA expression were equivalent to levels of Axin protein expression, with the exception of the TE8 line, in which Axin expression was reduced in comparison with protein expression (Figure 2B).

### Mutation of the *AXIN1* gene in oesophageal SCC

**Figure 3:**
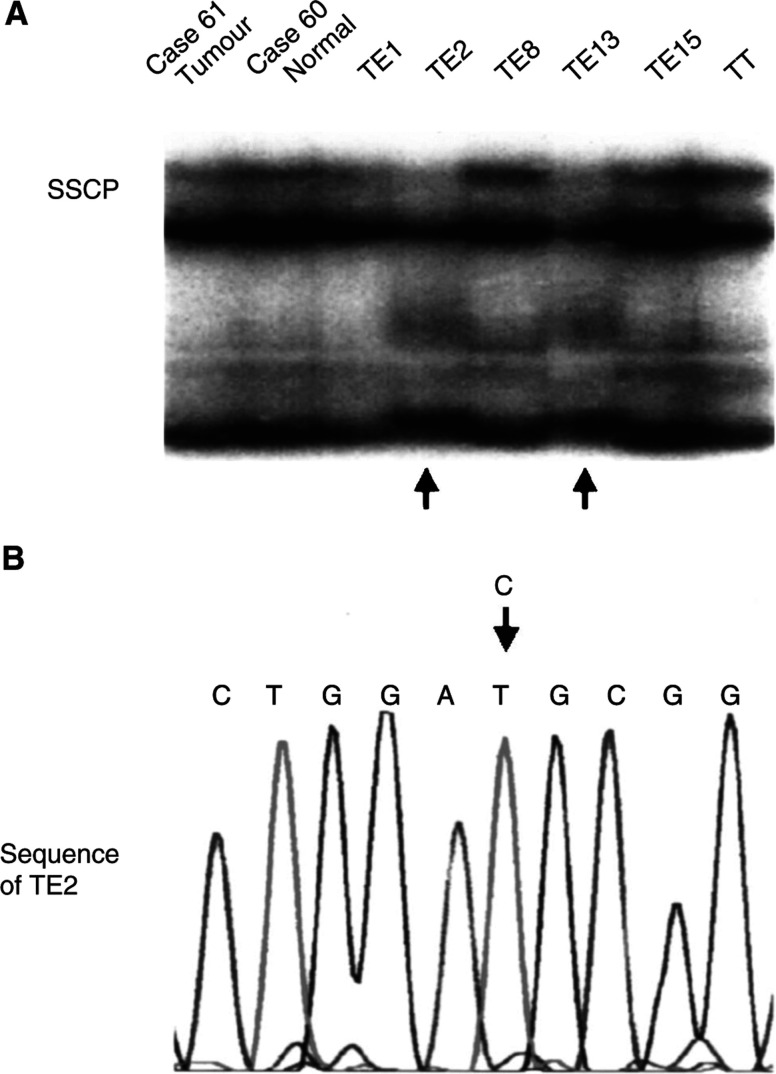
Mutation analysis of Axin. (**A**) Two aberrant bands of tumour DNA were detected in SSCP (arrow). (**B**) DNA sequencing of excised and reamplified DNA products of TE2 revealed C→T transition in codon 563 without amino-acid substitution. It was judged to be a silent SNP.

None of the 81 patients with oesophageal SCC had mutations, but five patients and three cell lines showed polymorphisms in the *AXIN1* gene (Figure 3

, Table 3

**Table 3:** Mutational analysis of Axin gene in oesophageal SCC

**Case**	**Age (year)/gender**	**Exon**	**Base change^a^**	**aa change**	**Codon**
30	66/male	4	G1256A	Yes (Arg→Cys)	419
40	54/male	4	G1256A	Yes (Arg→Cys)	419
13	64/female	5	G1396A	No (Ser→Ser)	485
15	62/male	5	G1396A	No (Ser→Ser)	485
17	59/male	5	G1396A	No (Ser→Ser)	485
TE1		5	G1396A	No (Ser→Ser)	485
TE2		5	C1690T	No (Asp→Asp)	563
TE13		5	C1690T	No (Asp→Asp)	563

a Amino-acid (aa) positions according to GenBank accession no. AF009674.

).

We confirmed three previously published single-nucleotide polymorphisms (SNPs) (Lin *et al*, 2000; Dahmen *et al*, 2001). One polymorphism resulted in an amino-acid substitution, and the others were silent SNPs. However, there was no novel polymorphism or silent mutation.

## DISCUSSION

Axin is a negative regulator of the Wnt signalling pathway. It accelerates phosphorylation and ubiquitination of *β*-catenin, thus inhibiting importation of *β*-catenin to the nucleus and controlling cell proliferation. Although an association of Axin with carcinogenesis has been reported in colon cancer cell lines (Webster *et al*, 2000), HCC (Satoh *et al*, 2000; Laurent-Puig *et al*, 2001) and medulloblastoma (Dahmen *et al*, 2001), to our knowledge there has been no report concerning oesophageal SCC. Therefore, we investigated the association between Axin expression and oesophageal SCC.

First, we performed an immunohistochemical study of the correlation between Axin expression and clinicopathologic factors in patients with oesophageal SCC. Axin expression was seen in the cytoplasm in normal oesophageal stratified squamous cells and tumour cells. In tumour tissues, Axin expression was inversely correlated with depth of invasion, lymph node metastasis, and lymphatic invasion. When we examined the Axin status of tumour cells that had invaded lymph vessels, their Axin expression was reduced or lost in most cases, suggesting that reduced expression of Axin initiates or promotes tumour progression. As Axin is a negative regulator of *β*-catenin/TCF-dependent cell proliferation (Kikuchi, 1999) and carcinogenesis (Barker *et al*, 2000), loss of Axin expression in oesophageal SCC may lead to tumour progression.

Western blotting revealed marked variation in the intensity of Axin expression, corresponding to the results of immunohistochemistry of the tumour tissues. During oesophageal carcinogenesis, some error may occur in the process of Axin protein production. Therefore, Northern blotting was performed to investigate the translation status of each tumour cell line. The status of RNA expression was variable, but the levels of Axin expression were equivalent to those of Axin protein with the exception of the TE8 cell line. That is, six of the cell lines – apart from the TE8 line – had no errors of translation. In these six lines, transcription errors might have occurred, because their intensity of Axin expression was weak compared with that in TE8. Thus, in the TE8 line, some errors might have occurred at the level of translation, or after. Further examination of this possibility will be needed.

Next, PCR–SSCP was performed to examine whether the variation of mRNA expression was derived from any genetic alterations of the GSK-3*β* or *β*-catenin binding site of Axin DNA. Five patients showed polymorphisms and three cell lines showed silent mutations in the *AXIN1* gene, but no pathogenetic gene mutation was detected. Although the frequency of *AXIN1* deletions in medulloblastoma is 12% (Dahmen *et al*, 2001) and a similar figure for genetic alterations has been demonstrated in HCC (Satoh *et al*, 2000), the results of our mutational analysis of oesophageal SCC were different, suggesting that association of *AXIN1* mutations with carcinogenesis is rare in oesophageal SCC. Similarly, one previous study detected no mutations in paediatric renal tumours (Miao *et al*, 2002). However, in addition to allelic losses, inactivation of transcription because of methylation in the promoter region could be responsible for downregulation of Axin. This possibility remains to be examined.

To examine whether Axin regulates only the Wnt-*β*-catenin-TCF/LEF pathway and determine which factors in this pathway would be good predictors of prognosis, we also analysed relations among Axin, *β*-catenin, and GSK-3*β* using immunohistochemistry and Western blotting. There was no significant association between either Axin and *β*-catenin, or between *β*-catenin and clinicopathologic factors (data not shown). Furuhashi *et al* (2001) have reported that Axin facilitates Smad3 activation in the TGF*β* signalling pathway. Ishiguro *et al* (2001) have reported that transcription of AXIN1 upregulated (*AXUD1*), a gene induced by *AXIN1*, is independent of the TCF/LEF complex and that *AXUD1* is frequently downregulated in some tumours. Oesophageal SCC may be regulated in a similar manner by an unknown pathway. GSK-3*β* expression was found to have no association with Axin expression or clinicopathologic factors (data not shown). Thus, there may be other pathways besides the Wnt signalling pathway that participate in carcinogenesis.

In HCC cells, adenovirus-mediated gene transfer of wild-type *AXIN1* induces apoptosis, regardless of the existence of *AXIN1* mutations (Satoh *et al*, 2000). Thus, transfer of wild-type Axin might offer a possible approach for gene therapy of oesophageal SCC.

In conclusion, Axin expression appears to be useful for predicting the prognosis of patients with oesophageal SCC, because Axin expression declines with tumour progression. Additional studies will no doubt elucidate the mechanism responsible for loss of Axin expression in tumour cells.
